# Crizotinib combined with bronchoscopic interventional treatment in ALK-positive inflammatory myofibroblastic tumor of left main stem bronchus: a case report

**DOI:** 10.1186/s13019-023-02427-7

**Published:** 2023-11-10

**Authors:** Peng Zhang, Chenchen Wang, Zechao Lv, Mingxiang Du, Ruixin Xu

**Affiliations:** 1 Department of Cardiothoracic Surgery, The 961st Hospital of Joint Logistics Support Force of PLA, Qiqihar, 230200 China; 2 Department of Emergency and Critical Care Medicine, The 961st Hospital of Joint Logistics Support Force of PLA, Qiqihar, 230200 China; 3 Department of Pathology, Changzheng Hospital, Naval Medical University, Shanghai, 200040 China; 4 Department of Cardiothoracic Surgery, The 960th Hospital of Joint Logistics Support Force of PLA, No. 25 Shifan Road, Tianqiao District, Jinan, 250031 People’s Republic of China

**Keywords:** Inflammatory myofibroblastic Tumor, Pulmonary (bronchial) IMT, Lung, Case report

## Abstract

**Background:**

Inflammatory myofibroblastic tumor (IMT), also known as an inflammatory pseudotumor, is a unique type of intermediate soft tissue tumor that commonly occurred in the lung. Its unclear etiology and cellular activity brought about the confusion not only in naming of it, but also in diagnosis and treatment.

**Case presentation:**

We reported the case of an 18-year-old male student who suffered from shortness of breath, chest tightness and chest pain. Chest computed tomography scan showed a spherical neoplasm blocking left main stem bronchus. After fiberoptic bronchoscopy procedure, the results of histopathological and immunohistochemical analysis indicated an IMT. The targeted next generation sequencing based genomic profiling of the tumor using formalin-fixed and paraffin embedded tissue was performed and a *EML4-ALK* fusion was detected. The patient began to receive Crizotinib, a *ALK* tyrosine kinase inhibitor, at a dose of 250 mg twice daily orally. The patient has recovered well after the operation, and no recurrence or metastasis has been found after 12 months’ follow-up.

**Conclusion:**

By means of the diagnosis and treatment of this case, the characteristics and therapies of IMT are illustrated. In addition, it also provides a reference for the therapeutic strategy of IMT in the future.

## Background

Inflammatory myofibroblastic tumor (IMT) is an uncommon mesenchymal tumor, that distinguished by prominent myofibroblastic spindle cells, with dense infiltration of plasma cells, lymphocytes, and eosinophils [1]. In the past, lack of sufficient research resulted in non-standard naming, including inflammatory pseudotumor, plasma cell granulomas, xanthogranuloma, fibrous histiocytoma, inflammatory fibrosarcoma and fibroxanthoma, of which inflammatory pseudotumor is most common used, however [2].

Until nearly two decades ago, IMT was defined as a low-grade malignant tumor, instead of benign tumor-like lesions that many scholars thought it as before. In spite of unclear etiology and natural course, IMT is observed to have biologic characteristics of invasion and metastasis. Lung is the most common location of IMT, however, no obvious clinical manifestations and atypical characters of radiologic signs make it difficult to early diagnose unless the pathological histology is performed [3].

In this paper, we report a case of IMT originating in the left main stem bronchus (LMB), aiming to review the imaging features, histopathological characteristics, treatment strategies and prognosis of this uncommon tumor. We present the following case in accordance with the CARE reporting checklist.

## Case presentation

The case was an 18-year-old male student with shortness of breath, chest pain and chest tightness for 1 day. The patient reported a history of smoking 20 cigarettes one day, on average, for one year.

**Fig. 1:**
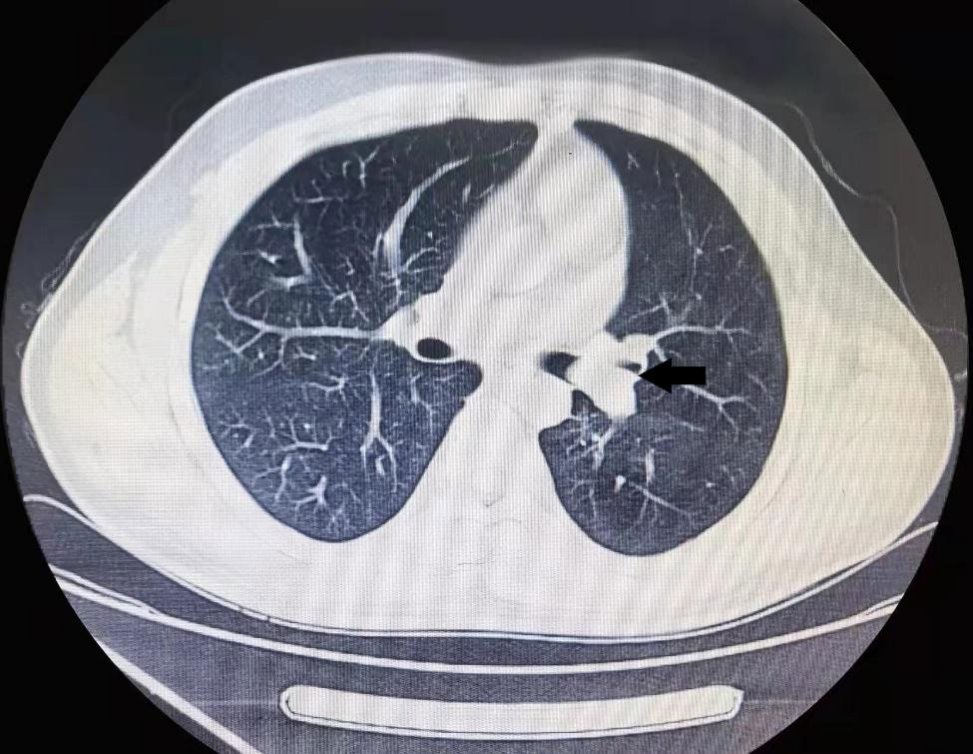
Chest CT revealed that the nodular shaped lesion with abundant blood supply within the LMB. The *arrow* indicates the lesion

**Fig. 2:**
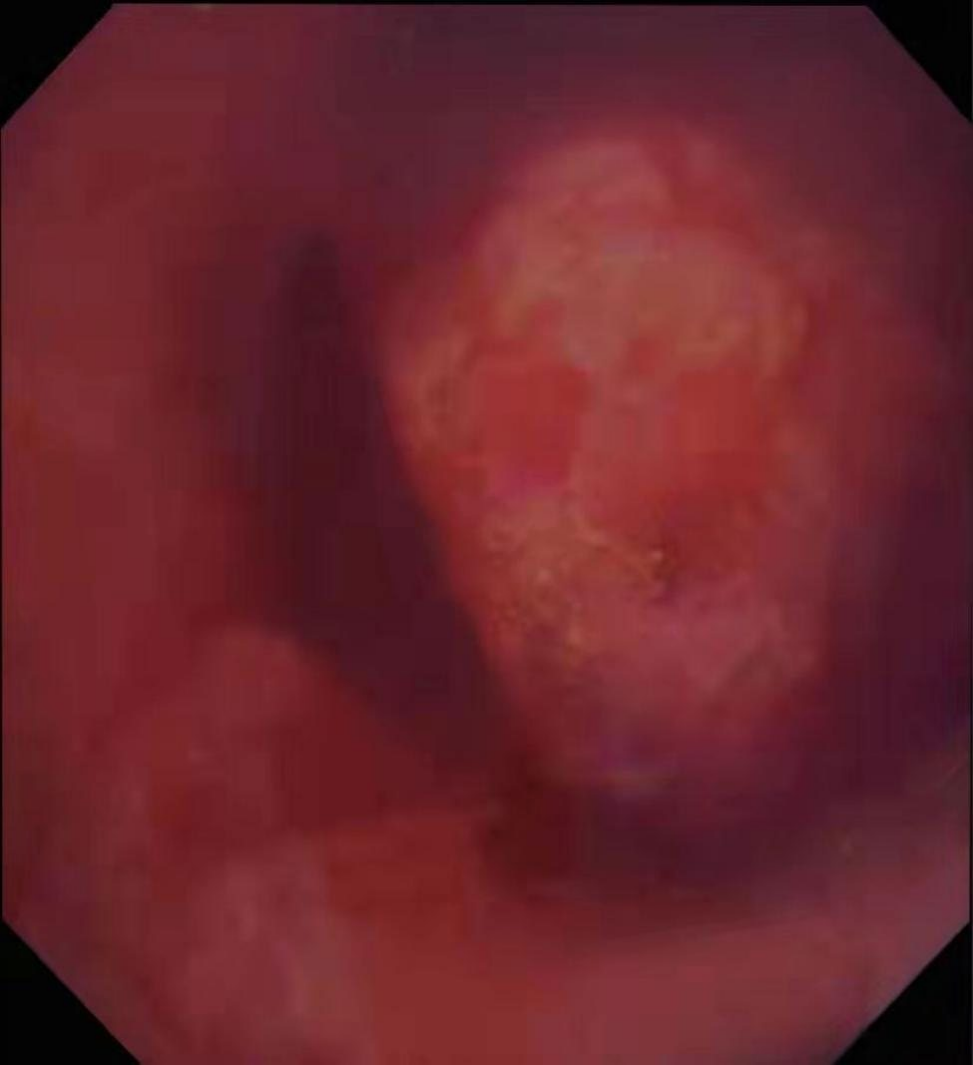
FOB showed that the opening of the the left main stem bronchus was normal, and a spherical new creature was seen blocking the lumen about 3 cm from the carina

Chest x-ray was normal. Chest computed tomography (CT) revealed that the lesion with abundant blood supply within the LMB, measuring 14 × 9 mm in size (Fig. 1). Pulmonary function testing revealed a restrictive pattern with reduced vital capacity to 1.44 L. There were no metastases on the bone scintigraphy, cranial magnetic resonance imaging and abdominal ultrasonography. Super selective angiography showed the tortuosity of branches of bronchial arterial, and the mass in blood supply area stained with contrast agent. In order to dismiss intraoperative bleeding and shrink the lesion, the supper selective bronchial arterial embolization was performed. Subsequent electron bronchoscopy under general anesthesia showed that the opening of the LMB was normal, and a spherical soft tissue mass was observed blocking the lumen about 3 cm from the carina (Fig. 2). The tissue mass was removed out through endoscopic polypectomy with the snare and alligator biopsy forceps. The base of the tumor was wide and there were still residual tissues. The argon plasma coagulation (APC) catheter was taken and inserted, and the end of APC electrode was 5 mm away from the residual tissues. With repeated multiple points of cauterization for 1 to 3 s each time, the coagulated tissues were removed by biopsy forceps continuously until all the residual tissues were cauterized and crusted. When no active bleeding was observed, the bronchoscope was withdrawn.

**Fig. 3:**
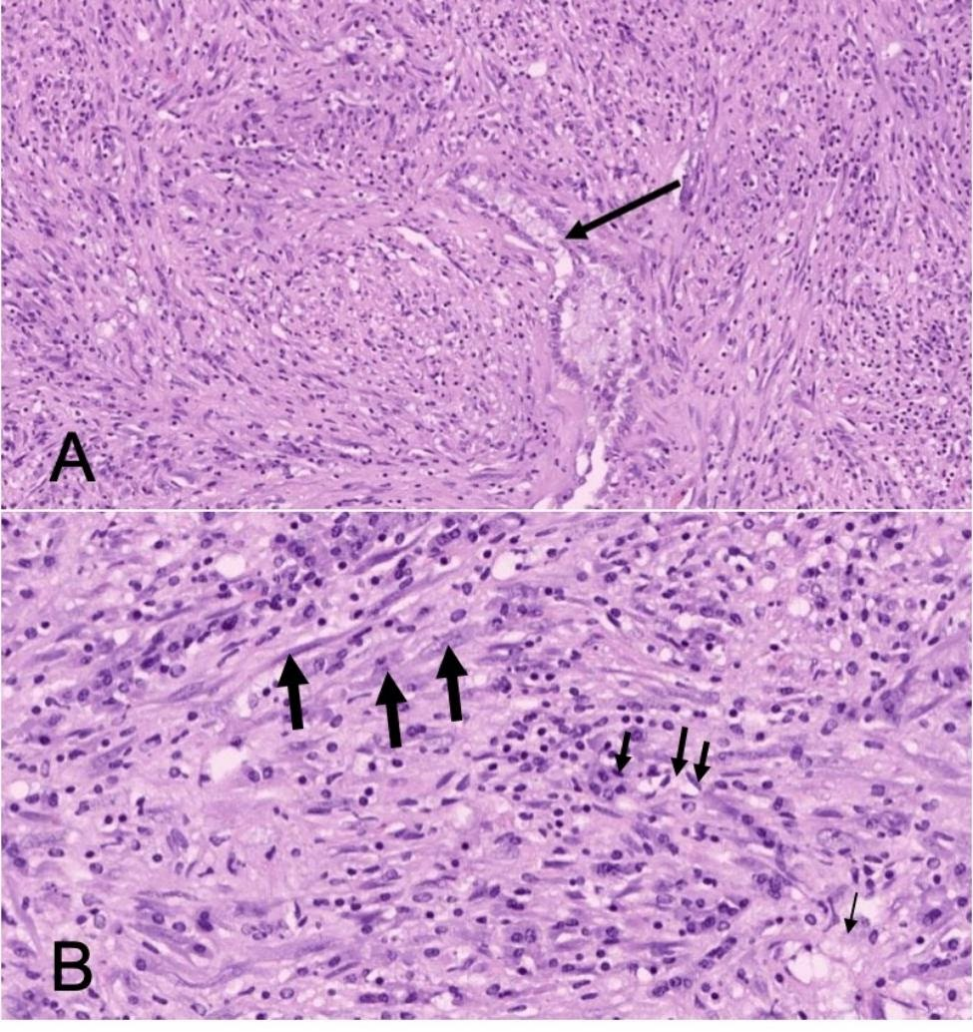
The tumor grows in a dense fascicular structure way, residual bronchiolar mucosa can be seen (*arrows*) (A). The tumor is mainly composed of spindle cells (*heavy arrows*), with scattered lymphocytic cells (*median arrows*) and foamy histiocytes cells (*thin arrows*) in some areas (B). Original magnification, ×100 (A) and ×400 (B)

**Fig. 4:**
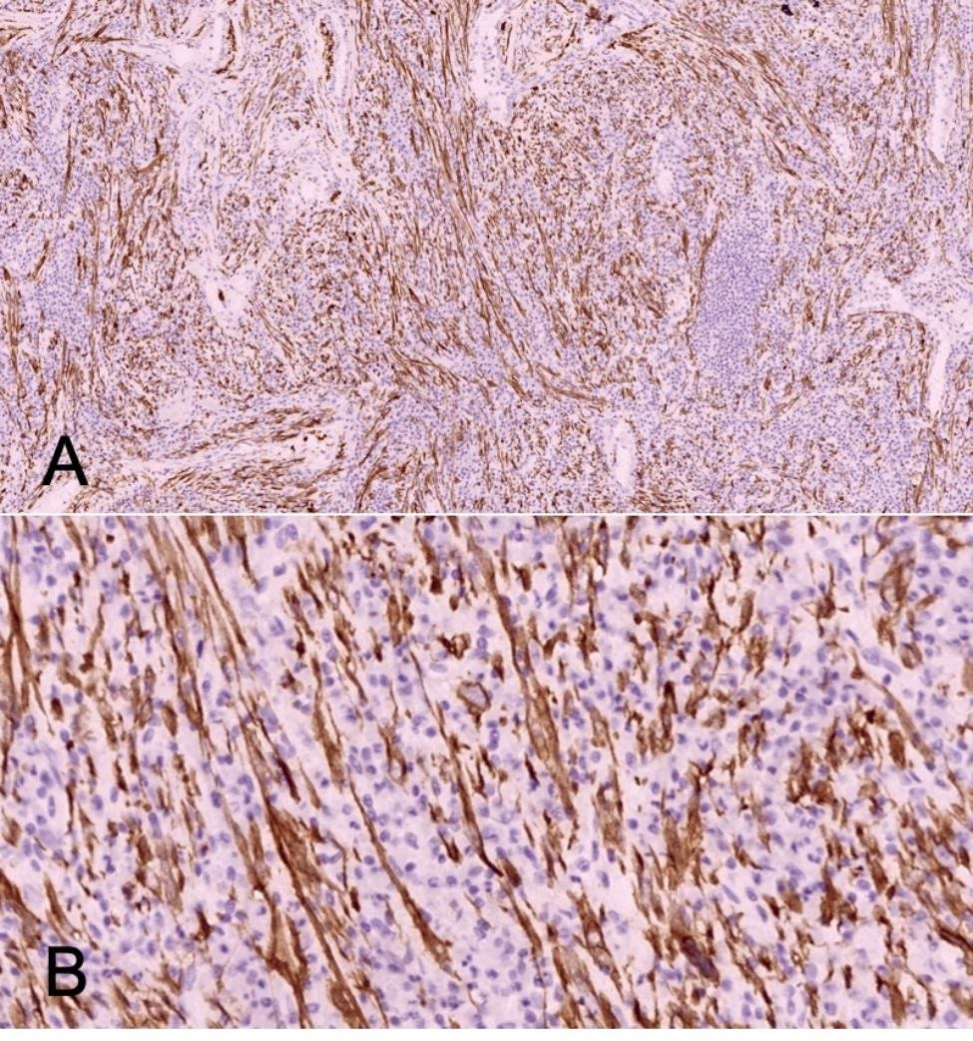
Immunohistochemical Staining shows positive for ALK. Original magnification, ×100 (A) and ×400 (B)

**Fig. 5:**
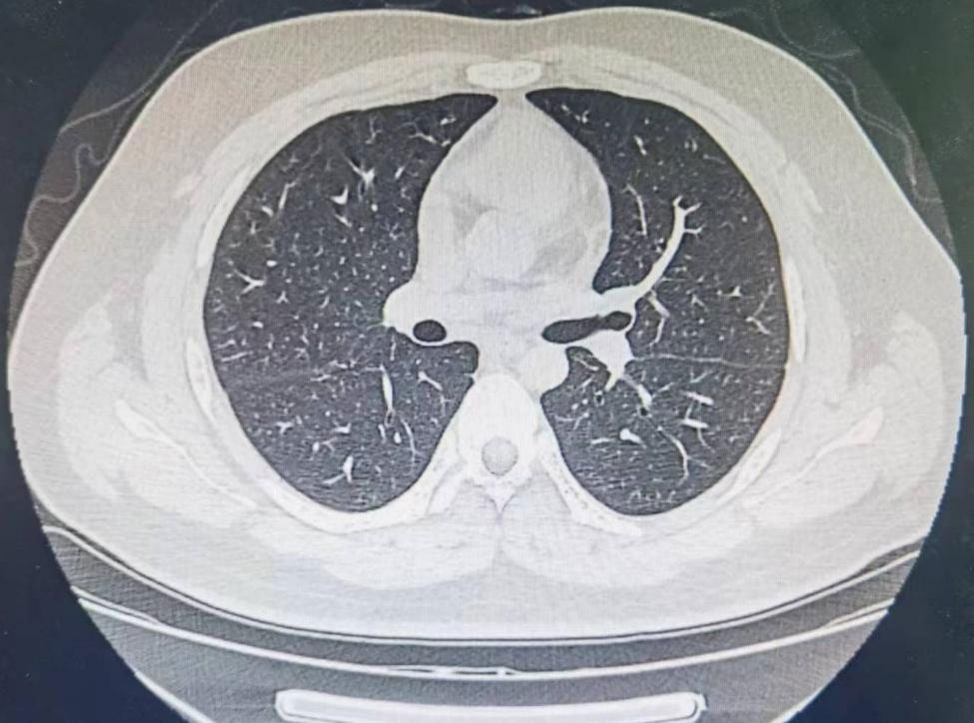
Postoperative chest CT showed patency of the left main bronchus

**Fig. 6:**
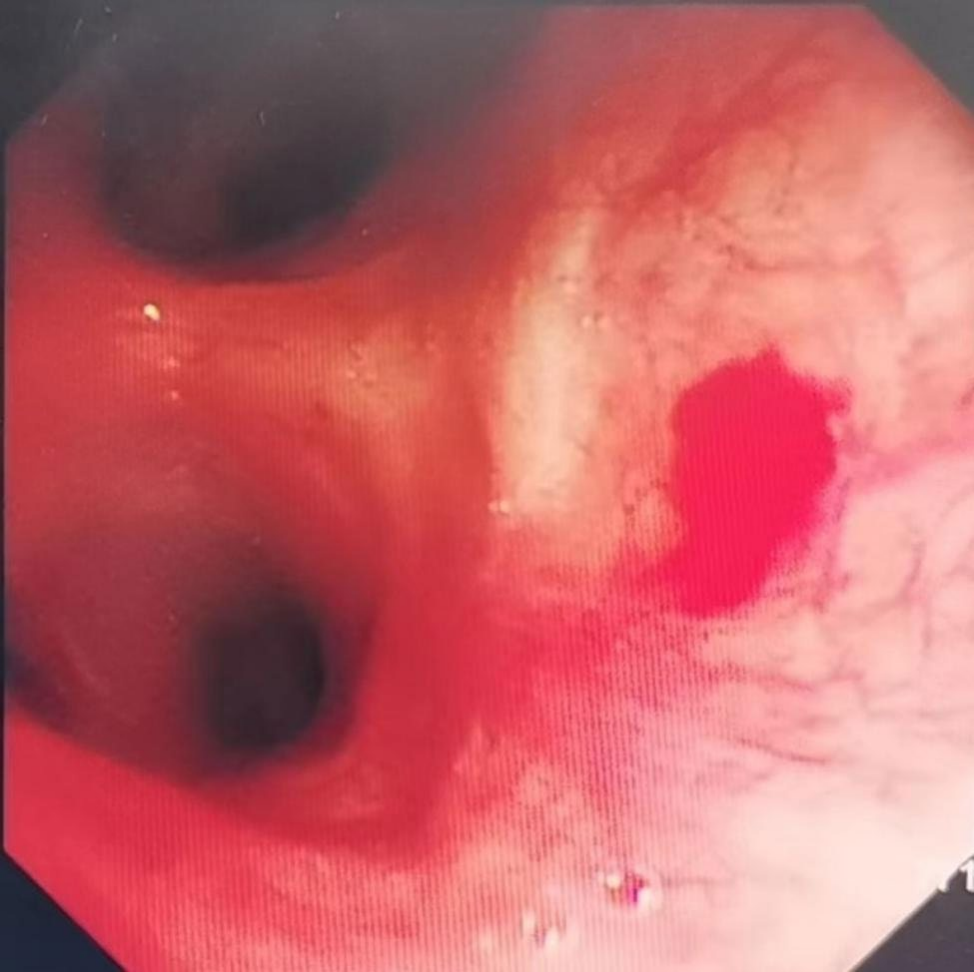
Postoperative bronchoscopy showed normal opening of the left main bronchus and postoperative scar at the distal end of the left main bronchus

Pathological diagnosis revealed that the IMT with low-grade malignancy of LMB may be possible. Under the epithelial mucosa of the respiratory tract, there were lots of spindle cell tumors consisting with scattered lymphocytes and foamy histiocytes cells infiltrated (Fig. 3). Immunohistochemical staining results as follows: *EMA* (-), *Vim* (+), *SMA* (-), *S-100* (-), *CD34* (-), *STAT-6* (-), *CD99* (-), *bcl-2* (-), *Ki67* (5%+), *SOX-10* (-), *ALK* (+), *Desmin* (-), *p63* (-), *FLI-1* (-), *HMB45* (-) *and Myo-D1* (-) (Fig. 4). The targeted next generation sequencing (NGS)-based genomic profiling of his tumor using formalin-fixed and paraffin embedded (FFPE) tissue was performed and a *EML4-ALK* fusion was detected. Although bronchial sleeve resection was recommended, the patient and his parents strongly rejected the further surgery, hence we proposed an individualized therapy that the patient began to receive Crizotinib, a *ALK* tyrosine kinase inhibitor, at a dose of 250 mg twice daily orally. The patient has recovered well after the operation, and no recurrence or metastasis has been found after 12 months’ follow-up (Figs. 5 and 6). Pulmonary function tests were measured at 3 and 6 months after interventional bronchoscopic resection, indicating the pulmonary function improved to 2.78 and 3.03 L, respectively. There were no serious side effects during Crizotinib treatment. At the beginning of treatment, however, the patient had a transient blurred vision and liver dysfunction, that the highest alanine aminotransferase was 210.6 U/L. With the extension of treatment time and the use of liver protection drugs, liver function gradually improved to normal level.

## Discussion

IMT, formerly referred to as one of inflammatory pseudo-tumours, is an infrequent disease, initially reported as a benign lung tumor in 1939. According to the published literatures, lung is the most common site of IMT, accounting for 0.7% of all lung tumors. Moreover, IMT mostly occurs in the lung parenchyma and rarely involve the bronchus. The case of tracheobronchial IMT is relatively rare.

### Aetiology of IMT

Currently, it is not absolutely clear about the aetiology and nosogenesis of IMT. It is regarded as a result of the anomalous reparative processes due to the stimulations of inflammation, trauma, surgical injury, human herpesvirus-8 [4, 5], Epstein-Barr virus infection [6, 7] etc. leading to proliferation and differentiation of myofibroblasts being the major elements for the composition of IMT.

Since many prior studies have shown that IMT is closely related to the rearrangement of the anaplastic lymphoma kinase (*ALK*) gene on 2p23 chromosome, the positivity of *ALK* expression by immunohistochemistry (IHC) contributes to the diagnosis of the disease [8]. Lovl et al. [9] analyzed 37 cases of IMT, 70% of which were *ALK* IHC positive. Up to now, genes fused with ALK in IMT demonstrated contain *SEC31A*, *TPM3*, *TPM4*, *TFG*, *CLTC*, *FN1*, *CARS*, *LMNA* and *PRKAR1A*. Abnormal structural recombination of the *ALK* gene can result in aberrant activation and expression of *ALK*, which induces the formation of a chimeric fusion protein, leading to the proliferation of myofibroblasts and hence promote tumorigenesis, progression and metastasis of IMT. Additionally, other genetic abnormalities are gradually confirmed, such as *YWHAE-ROS1*, *TFG-ROS1* and *NAB2-PDGFRβ* [9–11].

### Clinical and imaging features in IMT

The clinical manifestations of IMT are lack of specificity, depending on the site of origin and the effects of the mass. Most of pulmonary IMT might be asymptomatic due to peripheral tendency and slow growing, whereas, endobronchial and aggressive/larger parenchymal lesions can cause different symptoms, varying from chest pain, chest tightness, cough, wheeze, hemoptysis and dyspnea to pyrexia, weight loss and general fatigue. Of note, endobronchial lesions may cause neoplastic bronchiostenosi, leading to obstructive pneumonia with cough, sputum production, pyrexia and so on.

The imaging characteristics of IMT are often nonspecific. Chest X-ray is of limited help in confirmation for IMT owing to it poor sensitivity, though occasionally IMT can be detected as an incidental occurrence. The radiological findings of pulmonary IMT in plain chest CT scans commonly shows a soft tissue mass, with well-defined. After the administration of a contrast agent, the lesions present various degrees of enhancement or calcified regions [12]. Invasion to pulmonary hilar structures very often arises from central lesions, whereas, peripheral lesions tend to intrude into pleura. Magnetic resonance imaging (MRI) is considered to better obtain inner IMT tumor tissue and identify the lesion margin and the relationship of the lesion with vital surrounding organs and tissue structures. However, the application of MRI in pulmonary IMT is of limited, to a great extent, due to the pulmonary structural characteristics, hence few MRI findings have been reported so far. The lack of specificity on imaging features, as well as insufficient radiologic studies results in confusion about distinguishing it with rhabdomyosarcomas, lymphomas and teratomas [13].

### Diagnosis of IMT

For diagnosis of IMT, histopathology is considered as the gold standard. The follow latest criteria are formulated by world health organization (WHO) to diagnose IMT [14]: (I) compact or loose fascicles of spindle cells with a predominant infiltration of inflammation cells and a variable fibroid or mucoid stroma; (II) expression of *ALK* seen in up to 60% of cases. The desirable diagnostic criterion is *ALK* or other gene reassortments. On the basis of the distribution of myofibroblastic spindle cells, inflammatory cells, and infiltrating features, the pathological classifications of IMT are as follows: (I) a myxoid-vascular type, featuring a mass of immature neovascularization and mucus degeneration interstitial tissues with fascicles of spindle-shaped myofibroblasts and varying amounts of inflammatory cells; (II) a compact spindle cell type, featuring a large number of compact spindle cells with scattered inflammatory cells; and (III) a hypocellular fibrous type, featuring abundant collagen fibers with inclusion of the tumor cells. As is stated above, due to lack of specificity in clinical and imaging features, cytological, histological and immunohistochemical analysis is critical to diagnosis of the IMT.

Immunohistochemical analysis might be more beneficial to discriminate IMT and tumors with analogous histopathology, such as malignant fibrous histiocytoma, sarcomas, lymphomas and spindle cell carcinomas. The expression of *ALK* accounts for about 50% of cases of IMT, with the help of conventional IHC techniques [15]. Tan et al. [12] elucidate immunohistochemical findings of the 54 patients with IMT, that the positive rate of makers *ALK*, *SMA* and *Vim* were 44.4%, 88.9% and 87.0%. Of note, further studies on *ALK* IHC-negative cases showed next generation sequencing (NGS), fluorescence in situ hybridization (FISH) and intercalated antibody-enhanced polymer (iAEP) methods may detect novel *ALK* fusions [9, 16, 17]. The progression of highly sensitive and dependable detection means of *ALK* expression and reassortments will be crucial to not only improve diagnostic accuracy but also guide therapeutic strategies and prognostic conditions.

### Treatment of IMT

Complete surgical resection of the tumor is the preferred method for treatment of IMT. Also note that surgical strategy should be determined according to the patient’s general condition, size and location of the lesions. Since generally defined as a low-grade malignant tumor, the treatment of pulmonary IMT is similar to lung cancer. For larger lesions or high suspicion of malignant transformation, lobectomy with lymph node cleaning is recommended. As to smaller solitary pulmonary lesions, the specific surgical procedure should be determined based on the intraoperative frozen-section examination.

IMT involving the bronchus is even rarer. The case of the IMT of LMB by Privitera et al. [18] reported involved bronchial sleeve resection for complete removal of the lesion. Oztuna et al. [19] proposed that bronchoscopic resection is a viable alternative in cases of IMT restricted to the tracheobronchial tree. Our case showed that the IMT of LMB was removed out through endoscopic polypectomy with the snare and alligator biopsy forceps. Owing to *ALK* expression observed through IHC, the case presented in our study has been being treated with Crizotinib 250 mg orally once daily until now. As an oral small-molecule tyrosine kinase inhibitor, Crizotinib, targeting the *ALK*, *ROS1* and *MET* tyrosine kinases, is well approved to induce clinically significant responses in non-small cell lung cancer [20, 21]. Additionally, there have been case reports proving that therapy of some cases of *ALK*-positive IMT with Crizotinib, especially those in which surgery is contraindicated and cases of unresectable lesions, multifocal disease, or postoperative recurrence, could effectively inhibit tumor growth and decrease tumor size [22]. Corticosteroids might be of benefit to children in cases of hilar and mediastinal invasion, yet generally not helpful to adults [23].

### Prognosis of IMT

The current studies provide evidence that the tumor size and the quality of surgical resection are the major factor influencing prognosis of IMT. For solitary pulmonary nodules, complete surgical resection confers a favorable 5 and 10-year survival in these patients of 91% and 77.7%, respectively [1]. The tumor size of less than or equal 3 cm is correlated with improved survival [23]. Recurrence rates following complete surgical resection is only 2%, compared to 60% of incomplete resection. Recurrence may occur several years after the initial diagnosis, which underscores the necessity of long-term follow-up, especially those with incomplete resection. The probability of metastatic spread of IMT is less than 5%. To the best of our knowledge, this is the first case that was treated with interventional bronchoscopic resection, followed by medical therapy with Crizotinib orally. The patient has been followed-up 12 months without recurrence or distant metastasis.

## Conclusion

To sum up, IMT represents an exceedingly rare low-grade malignancy, which most frequently occurs in the lung. Involvement of the bronchus is an even more rare occurrence. Due to the lack of typical clinical manifestations or particular imaging characteristics, diagnosis of IMT primarily depends on histopathology. Although it is generally recommended to perform complete surgical procedures, the combination of endoscopic resection and small-molecule tyrosine kinase inhibitor may be an appropriate oncologic procedure as for a completely endoluminal mass, as in this case.

## Data Availability

The datasets used during the current study are available from the corresponding author on reasonable request.
